# Comment on “Incidence of Type 1 Diabetes among Children and Adolescents in Italy between 2009 and 2013: The Role of a Regional Childhood Diabetes Registry”

**DOI:** 10.1155/2016/6302508

**Published:** 2016-11-22

**Authors:** Paola Ballotari, Valeria Manicardi, Paolo Giorgi Rossi

**Affiliations:** ^1^Interinstitutional Epidemiology Unit, Local Health Authority of Reggio Emilia, Via Amendola 2, 42122 Reggio Emilia, Italy; ^2^Arcispedale Santa Maria Nuova-IRCCS, Viale Umberto I 50, 42123 Reggio Emilia, Italy; ^3^Internal Medicine Department, Montecchio Hospital, Local Health Authority of Reggio Emilia, Via Barilla 16, 42027 Montecchio, Italy

We read the article by Fortunato et al. [1] with great interest. In this study, one of the aims was to evaluate the incidence of Type 1 Diabetes (T1DM) in subjects <18 years of age in Apulia (Italy) by using three data sources: a hospital discharge registry, user fee exemption registry, and drug prescription registry. The authors found a progressive decrease in the annual incidence rate over the study period (2009–2013). According to previous studies, the incidence rate of Type 1 Diabetes among children (0–14 years of age) had been increasing [2–5] over the past 20 years. Moreover, a similar study carried out in Friuli-Venezia Giulia (a region in northern Italy) [6] found an almost stable incidence over the same period, excluding the first year of registration (2010). In the province of Reggio Emilia (Italy), where the diabetes register has been collecting data since 2009 [7], the trend of incidence is stable. Despite actions performed to clean up the Apulian data, residual misclassification of prevalent cases may have affected the data. Indeed, given the absence of a clinical database where the date of diagnosis is recorded, some prevalent cases may have been misclassified as incident cases; however, such misclassification declines after some years of registration because the undetected prevalent cases diminish. Therefore, the final effect of this declining bias is a false decrease of incident cases in the initial years of registration.  Table 1, which is based on our data, reports the difference in incidence rate calculated using the date of diagnosis and the date of entry from the linkage of the data sources. A highly interesting approach for the Apulian register could be to broaden the network of pediatricians and/or to include hospital pediatric units in the same network, given their role in managing T1DM in children and adolescents. In this way, the register would merge clinical and routinely collected databases, thus enhancing completeness and reliability.

## Figures and Tables

**Table 1:** T1DM incidence rates (per 100,000) among subjects, 0–18 years of age, in Reggio Emilia province, 2010–2013, by date of diagnosis and by date of entry.

Year	Population	By date of diagnosis			By date of entry		
		Cases	Rate	95% CI	Cases	Rate	95% CI
2009	95625	16	16.7	9.5–27.2	21	22.0	13.6–33.6
2010	97798	18	18.4	10.9–29.1	21	21.5	13.3–32.8
2011	99217	16	16.1	9.2–26.2	15	15.1	8.5–24.9
2012	97473	19	19.5	11.2–30.4	18	18.5	10.9–29.8
2013	99249	17	17.1	10.0–27.4	19	19.1	11.5–29.9
2014	100815	19	18.8	11.3–29.4	19	18.8	11.3–29.4
